# Political economy of adolescent mental health and well-being in Sweden: how to overcome barriers to effective financing and youth-centered collective action

**DOI:** 10.1186/s12889-025-22737-w

**Published:** 2025-04-23

**Authors:** Olivia Biermann, Mariam Claeson, Mirjam Derko, Carolina Mikaelsdotter, Alma Nordenstam, Stefan Swartling Peterson

**Affiliations:** 1 Department of Global Public Health, Karolinska Institutet, Tomtebodavägen 18a, Stockholm, 17177 Sweden; 2 Makerere University School of Public Health, Kampala, Uganda; 3 Department of Women’s Children’s Health, Uppsala University, Uppsala, Sweden

**Keywords:** Political economy analysis, Adolescent mental health and well-being, Youth-centered services, Collective action, Sweden

## Abstract

**Background:**

In Sweden, adolescents (10-19-year-olds) increasingly face problems related to their mental health and well-being, driving the rise in rates of mental ill-health (e.g., anxiety, depression, suicidal ideation and self-harm), and care-seeking for psychiatric conditions. Although awareness about adolescent mental health and well-being (AMH) has grown in recent years, this has not translated into effective financing and youth-centered collective action to change the trajectory. This study investigates the barriers to financing and action for AMH in Sweden, drawing lessons for global learning.

**Methods:**

This study triangulates data from interviews, focus group discussions (FGDs), consultation and document review. The interviews and the consultation included stakeholders who have experience with and knowledge of AMH, e.g., government, civil society and funders. The FGDs included youth representatives who are mental health advocates, some with lived experience of mental ill-health. We collected the qualitative data between February 2022 and October 2023, analyzing the data using a political science framework and thematic analysis.

**Results:**

The themes that this study identifies highlight the barriers to effective financing and youth-centered collective action: (1) the limited data and evidence related to AMH; (2) the divergent definitions of and ways of framing AMH; (3) the growing but fragmented AMH stakeholder landscape; and (4) the weak multidisciplinary and multisector collaboration for AMH.

**Conclusions:**

Transformational change is needed to improve AMH outcomes through effective financing across sectors in support of youth-centred collective action. Identified barriers may be overcome by: researchers focusing on advancing data and evidence, especially on what works in AMH prevention and promotion; stakeholders, especially advocates of AMH, deliberating and agreeing on a broadened definition and framing of AMH, led by youth and using a positive narrative; decision-makers, funders and researchers strengthening leadership, accountability and adolescent engagement for AMH, and enhancing multidisciplinary and multisector collaboration for AMH, backed up by well-coordinated, youth-centered health and social services, learning from good practices within and outside of Sweden.

## Background

In Sweden, adolescents (10-19-year-olds) face increases in mental ill-health, including depression, anxiety, suicidal ideation and self-harm, with a steady rise in care-seeking for psychiatric conditions [1–3]. Among boys in Sweden, self-harm accounts for a larger proportion of the burden of disease than depression and anxiety while the pattern is the opposite in girls [2]. Suicide is the fourth-leading cause of death in girls and boys in this age group [4], and self-reported suicidal ideation through helplines has significantly increased from 2018 to 2022 [3]. Compared to other Nordic countries, the increase in psychosomatic symptoms among adolescents has been more extensive in Sweden [5].

Determinants of adolescent mental health and well-being (AMH) in Sweden range from factors within the family and the family’s socio-economic conditions, the school and learning environment, to societal changes such as increased individualization, and openness about mental ill-health [2]. Sweden has the highest rate of children living in relative poverty among Nordic countries, and ranks 22nd out of 39 assessed rich countries, with nearly one in five children (18%) living in relative poverty [6].

The Swedish society faces a large burden of AMH-related challenges, similar to other countries across all income levels [7]. In recent years, a growing evidence base has highlighted the need for multisector and multidisciplinary action to address the many drivers of AMH globally; these include legal and regulatory interventions at the population level, education and support for caregivers, and environmental and psychosocial support at adolescent gathering spaces [8, 9].

The government, research councils, and foundations have invested in AMH in Sweden, and several policies relevant for mental health and well-being have been published in recent decades. In 2020, the Swedish government commissioned a proposal for a future strategy for mental health and suicide prevention. The proposal was published in 2023, underscoring the need for increased investments in children and adolescents [10]. In 2024, the government announced additional investments in this area [11] and published a national strategy for mental health and suicide prevention [12] and an accompanying action plan [13]. Still, these efforts appear insufficient, responses siloed, and actors fragmented, as investments in AMH have yet to translate into multidisciplinary and multisector youth-centered collective action to drive transformational change.

This study investigates the barriers shaping AMH financing and action, and explores the opportunities for policy reform and the translation of policies into more effective, efficient, and sustainable financing for AMH, driven by youth-centered collective action.

## Methods

This study triangulates data from interviews with key-informants with experience and knowledge in AMH; focus group discussions (FGDs) with youth representatives who are mental health advocates, some with lived experience of mental ill-health; consultation with key stakeholders and document review. We collected the qualitative data between February 2022 and October 2023, and analyzed the data using a political science framework by Shiffman [14] and applying thematic analysis by Braun et al. [15]. We reported the qualitative data collection using the consolidated criteria for reporting qualitative research [16] (Additional file 1).

### Study setting

Sweden has a population of about 10.6 million out of whom 1.2 million (11%) are 10–19 years old [17]. The central government manages the national budget and provides directives, while the mandate for AMH implementation is split between regions and communes. Regions are responsible for provision of healthcare services, including primary health care, and child and adolescent psychiatry (*barn- och ungdomspsykiatrin*). Communes oversee social services and schools, including school health services, which provide medical, psychological and psychosocial interventions [18]. Both regions and communes separately or jointly run youth clinics (*ungdomsmottagningar*) [19].

### Conceptual underpinning

**Fig. 1:**
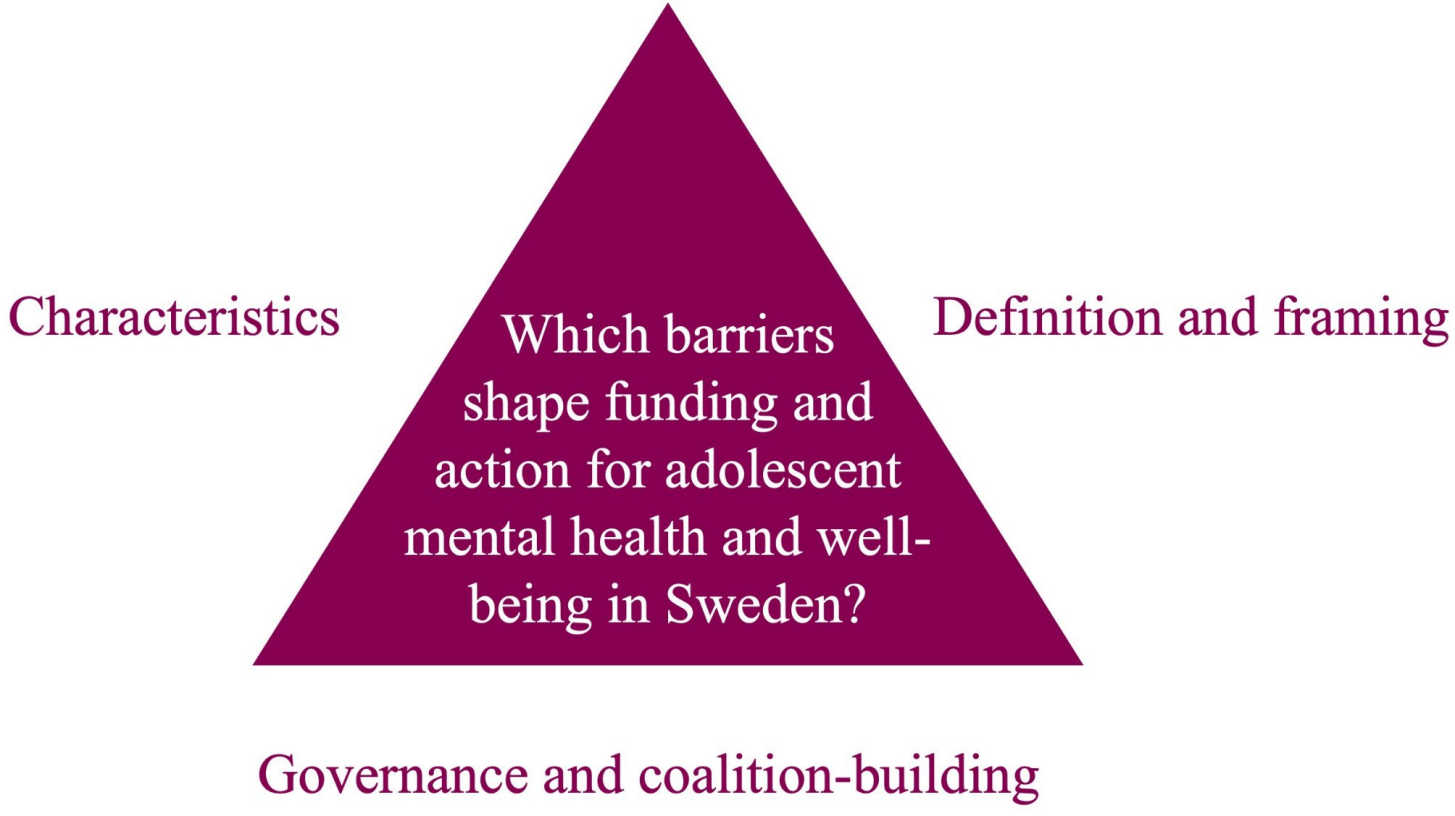
Analytical framework adapted from Shiffman [14]

Our analytical framework (Fig. 1) is adapted from Shiffman [14], considering: (1) *issue characteristics* [20, 21]: the features of the problem which influence how easy or difficult is it for stakeholders to address it (e.g., if an issue is stigmatized it may be more difficult to tackle); (2) *problem definition* [14, 22]: generating consensus among stakeholders and potential allies on what the problem is and how it should be addressed, and *framing* [14, 23]: portraying the issue in ways that help to prioritize it and inspire collective action; as well as (3) *governance* [14, 24]: establishing institutions and accountability mechanisms to facilitate collective action within a given structural and administrative context, and (4) *coalition-building* [14]: forging alliances across sectors.

### Document review

We reviewed the grey literature, identifying key policy documents and reports. The review did not aim to be comprehensive but to identify key documents for triangulation with the findings from the interviews and FGDs. We used Google and hand-searched websites of Swedish government agencies, civil society organizations, associations, funders, and networks. We applied search terms (in Swedish) such as “adolescent”, “adolescence”, “youth”, “young people” and “mental health”, searching for documents in Swedish without any set timeframe. We identified 66 documents in the grey literature, published between 1 January 2004 and 12 January 2025, and extracted data in the form of text passages related to issue characteristics, problem definition and framing, governance and coalition-building into Excel. A selection of key policy documents and reports is included in Additional file 2.

### Interviews, FGDs and consultation

We used purposive and snowball sampling to identify key-informants, aiming to include multidisciplinary stakeholders working with AMH based at a broad range of organizations. We compiled an initial list based on our knowledge of experts and recommendations by colleagues in the field. We also used purposive and snowball sampling to identify youth representatives for the FGDs. Our inclusion criteria were youth representatives who are mental health advocates aged 15–19. We reached out to youth-led organizations and organizations with youth representatives, and contacted potential participants via e-mail. The snowball sampling led us to potential FGD participants up to 22 years of age who we decided to include, as they had started advocating for AMH as adolescents, making us interested in their experience.

We collected the data using semi-structured interviews and FGDs in Swedish via Zoom after obtaining written informed consent. We adapted the topic guides for the interviews and FGDs based on previous work [25], covering the different concepts in Fig. 1 above (Additional file 3, in English and Swedish). OB (a postdoctoral researcher with extensive experience in qualitative research) approached 37 potential interviewees of whom 17 were not included in the study; 14 did not respond, three did not have time or were not interested in participating. In total, 25 key-informants (18 female, 7 male) were interviewed. The interviews took on average 47 min (30–67 min). For the FGDs, OB approached 22 potential participants of whom twelve were not included in the FGDs; seven did not respond and five did not have time or were not interested in participating. Finally, OB and AN (a medical student with experience in youth advocacy) co-facilitated three FGDs with a total of 10 participants (5 female, 5 male). Three participants were 17, 19 and 20 years old respectively, and one was 22. The discussions lasted on average 62 min (50–80 min). At least two participants explicitly mentioned their own lived experience of mental ill-health during the FGDs. The interviews and FGDs were audio-recorded and professionally transcribed. All participants were offered to review the transcript of their respective interview/FGD, which only one interviewee requested. We determined for this sample to have sufficient information power to reach the study’s objective [26].

To review the preliminary findings and seek additional input, we conducted an in-person, three-hour consultation at Karolinska Institutet in Stockholm in October 2023. We included participants based in Stockholm, comprising individuals working with AMH based at a broad range of organizations, as well as youth representatives. We held the consultation with 27 participants (20 female, 7 male), including six individuals who had participated in the interviews or FGDs previously. OB, CM and MC took informal field notes, which we used for triangulation. Table 1 provides an overview of the 25 interviewees, 10 FGD participants and 27 consultation participants.

### Data analysis

We analyzed the data from interviews and FGDs using thematic analysis as described by Braun et al. [15], triangulating the findings with data from document review and consultation. We took the following analytical steps in English: *First*, OB and MD coded the qualitative data from interviews and FGDs using NVivo 14 software. *Second*, OB and MD categorized the codes based on the concepts in Fig. 1. *Third*, OB analyzed and summarized the preliminary findings with input from MC, MD and AN. *Fourth*, OB, MC, CM, AN and SSP discussed the preliminary findings during the consultation while taking informal notes, especially of opportunities highlighted. The notes were not formally analyzed in NVivo. *Fifth*, OB and CM compared the findings from the qualitative data with the findings from the document review and consultation for triangulation. *Sixth*, OB and MC identified the themes from the data. *Seventh*, OB and MC developed a first draft of the manuscript. All co-authors contributed to the final draft.

**Table 1:** Interviewees’ organizational affiliation and sex

	Interview	FGD	Consultation	Interview	FGD	Consultation	Total interviews	Total FGD	Total consultation	Total overall
	Female (n)			Male (n)						
**Total participants**	**18**	**5**	**20**	**7**	**5**	**7**	**25**	**10**	**27**	**62**
**Organizational affiliation**										
Government (national)	0	0	0	1	0	0	1	0	0	1
Government (regional)	2	0	0	0	0	1	2	0	1	3
Government (commune)	1	0	2	1	0	0	2	0	2	4
Government agency	1	0	4	1	0	0	2	0	4	6
University/research institute	2	0	0	3	0	0	5	0	0	5
Civil society organization	6	0	3	1	0	2	7	0	5	12
Professional association	1	0	0	0	0	0	1	0	0	1
Private sector	1	0	0	0	0	0	1	0	0	1
Health care provider	1	0	1	0	0	0	1	0	1	2
International organization	1	0	1	0	0	0	1	0	1	2
Research councils and foundations	1	0	4	0	0	1	1	0	5	6
AMH advocate with lived experience	1	0	0	0	0	0	1	0	0	1
Movements and networks	0	0	2	0	0	0	0	0	2	2
Youth representatives	0	5	3	0	5	3	0	10	6	16

## Results

To set the stage, we first describe how the attention to AMH in Sweden has increased without translation into effective financing and youth-centered collective action. We then elaborate on four themes, mapped onto the analytical framework, which illustrate the barriers for the latter.

*“There is a difference between making a problem visible and actually taking concrete measures against it. […] It is us young people who talk about our mental health or mental illness ourselves*,* while those who have the power to change are still the older generation who may not make it [AMH] visible and who may not know as much or prioritize it as highly.”* (Adolescent, FGD 2).

Attention to AMH has increased in Sweden over the past decades, according to the respondents. Adolescents, citizens, influencers, the media, and politicians talk more about AMH and debate the topic in public fora. The attention to AMH has been amplified by the coronavirus disease 2019 (COVID-19), the climate crisis, and the war in Ukraine. Moreover, large advocacy efforts have contributed to increasing the attention to AMH, such as the renaming of the Stockholm Globe Arena to “Avicii Arena” in honor of the late Swedish disc jockey Tim Bergling, alias Avicii, who died by suicide in 2018 [27].

However, the increased attention has not translated into sufficient resources and effective financing, as one respondent highlighted: *“there is money in the system*,* but it is misallocated and not used properly”* (Interview 18, female, regional government). The government funding of child and adolescent psychiatry were often mentioned as an indication of the high level of priority given to AMH in Sweden. However, these investments were mostly said to be short-term, neglecting comprehensive first-line care, prevention and promotion. Furthermore, the respondents described the financing landscape as challenging to navigate, especially for civil society organizations (CSOs), some of whom qualify for government funding while others “*fall between chairs*” (Interview 16, male, academia). The need to include AMH in government budget allocations across sectors, and to use data and evidence to inform prioritization and financing decisions, were emphasized.

*“You have a political logic that is about showing short-term victories that are easy to sell to the media and the public. In reality*,* […] they are not designed to have an effect. […] This has characterized Swedish mental health policy for 20 years; that quite a few billions have been spent over the years*,* but this has often taken place in the form of projects or temporary investments*,* and it has not helped to build up structures*,* strengthen capacity*,* work with prevention*,* promotion and early interventions.”* (Interview 24, male, national government).

The Swedish national strategy for mental health and suicide prevention launched in January 2025 highlights “increased investments in children and adolescents for good mental health throughout life” as one of seven goals [12]. To change the trajectory of AMH outcomes, there is not only a need for *increased* investments, but for a *better distribution* of resources within and across sectors, and evidence-based allocation across the mental health continuum targeting mental health and well-being; from mental health distress to mental disorders and psychosocial disability [28].

**Fig. 2:**
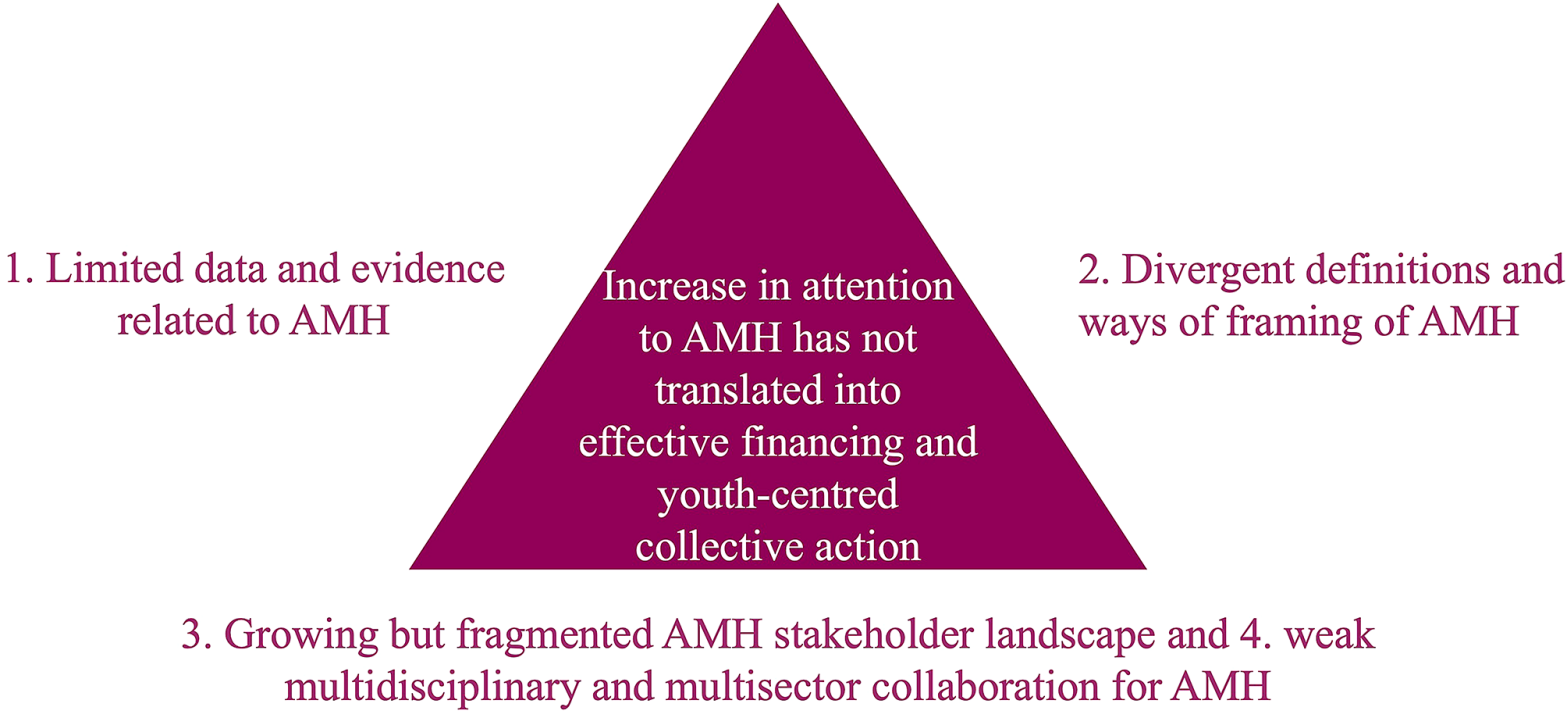
Overview of identified themes that highlight the barriers to effective financing and youth-centred collective action

The following four themes illustrate the main barriers to effective financing and youth-centered collective action in Sweden. Figure 2 provides an overview of the themes.

### Theme 1. limited data and evidence related to AMH

*“We think in Sweden […] that we have a lot of data. We don’t. We don’t have nearly as much data as we would need*,* and it is not coordinated in such a way that you can make good use of it.”* (Interview 13, female, national association).

Knowledge about AMH is increasing in the general population in Sweden, while evidence and data on trends in AMH outcomes at the population level are still scarce, the respondents said. The lack of data and evidence on what works in AMH prevention and promotion was perceived as a major barrier for scaling up action. Few initiatives were said to be monitored independently and evaluated for changes in AMH outcomes. The respondents pointed out that future AMH prevention and promotion interventions in Sweden, and related implementation research, would require differential approaches and disaggregated data, considering the influence of the many contextual factors such as income inequality, residence and migration status, violence, racism, social media, culture, and climate change. The document review confirmed that the possibilities of tracking AMH outcomes at the national level are limited, especially among vulnerable groups (e.g., adolescents with disabilities, those who identify as lesbian, gay, bisexual, transgender, queer, intersex, and asexual [LGBTQIA+] and national minorities) [10].

According to the respondents, opportunities for improved data and generation of evidence on AMH include: the use of available data generated from different sources including health and social services, and schools; the implementation of tools like the United Nations Children’s Fund’s (UNICEF’s) Measuring Mental Health Among Adolescents and Young People at the Population Level (MMAPP) [29]; the availability of implementation research funding for AMH; and, holding stakeholders accountable for their obligation to report. The consultation participants emphasized the opportunity to improve the “toolbox” for AMH data and evidence, and to develop and use standardized indicators, including those that can pick up early signs of mental health challenges. Furthermore, a document highlighted the opportunity for data and evidence to be coproduced with adolescents [30, 31], e.g., tracking the trends in AMH outcomes, including anxiety, depression, self-harm, and suicidal ideation, and evaluating the effectiveness of programs and interventions that are being introduced.

Among the challenges in monitoring and evaluating trends in AMH outcomes are the reliance on self-reported data, limited standardization and quality of AMH measuring tools, low response to surveys and participation rates in clinical studies and pre-formulated questions with little room to express individual experiences. During the FGDs, adolescents discussed legal barriers that, for example, limit the age of respondents and type of data that can be collected from adolescents without parental consent, and data sharing between agencies.

### Theme 2. divergent definitions of and ways of framing AMH

*”Mental ill-health is what’s talked about most. I like to put more weight on mental health rather than mental ill-health– to work preventively and to work broader*,* too*,* so that we’re not just there putting out fires.”* (Adolescent, FGD 2).

According to the respondents, various ways of defining and framing AMH exist. Some definitions focus on adolescent mental ill-health, defined as a clinical problem that requires a medical diagnosis and prompts a curative solution. Other definitions consider AMH as an integral part of a continuum of health and well-being, which was often seen as more conducive to prevention, promotion, and collective action. The document review showed that many reports talk about AMH as a clinical problem, while more recent reports increasingly refer to the strengthening of AMH as a resource for development [10, 32]. Similarly, a report from the Organisation for Economic Cooperation and Development [33] states that “momentum has been building in recent years to shift the conversation away from the sole prevention of mental ill-health and onto the promotion of positive mental health.” During the consultation, the participants confirmed that the narrative needs to shift from mental ill-health to mental health, and that adolescents want to own their narrative. They recommended using a positive narrative that highlights the benefits of investing in AMH across the mental health continuum [28]. The respondents did not consider the different narratives mutually exclusive.

The public debate about AMH has moved from being “*too alarming*” to being more nuanced and “*mature*”, e.g., in terms of focusing increasingly on the social determinants of and systems for AMH, according to one respondent (Interview 6, female, government agency). Another respondent described that since AMH has become a more “*fashionable*” topic there is a risk of “*bluewashing*” AMH in the public discourse, similar to the greenwashing of climate change in the context of the environmental debate (Interview 24, male, national government).

The current AMH narratives have been influenced by several factors, the respondents said. Stakeholders who have shaped the narratives include government agencies, (social) media influencers and adolescents who speak up about their lived experience. The common language around mental health has become more nuanced in Sweden, e.g., by the provision of clear definitions of relevant terminology (mental health, mental well-being, mental ill-health, mental problems and psychiatric conditions) by the Public Health Agency of Sweden, the National Board for Health and Welfare, and the Swedish Association of Local Authorities and Regions National Board for Health and Welfare [34]. During the FGDs, adolescents stated that the current narratives have also been influenced by a decrease in stigma directed towards adolescents with mental health challenges such as depression and anxiety, while suicidal ideation and suicide remain stigmatized.

### Theme 3. growing but fragmented AMH stakeholder landscape

*“It [addressing AMH] takes quite dedicated leadership I think– knowledgeable*,* so that you know the different systems and the different legislations*,* but also that you really want to invest the energy that will be needed to implement this.”* (Interview 13, female, national association).

An increasing number of stakeholders across disciplines and sectors are involved in AMH, according to the respondents. Key stakeholders who were mentioned included adolescents and their families; government and government agencies; different types of organizations and networks; the private sector; schools; health and social services providers; foundations and charities; research councils; (social) media and influencers (Additional file 4). The respondents often described the stakeholder landscape as fragmented with few stakeholders who have AMH as their sole or main focus.

Leadership and accountability were perceived lacking, by respondents and consultation participants alike, especially among those in positions of power. The latter caused limited collective action and lack of coordination across national, regional and commune levels which have different complementary mandates for AMH. The respondents suggested the development of a national AMH plan, with AMH explicitly reflected in budget allocations across sectors and administrative levels, and clarity on roles, mandates and lines of accountability. The document review similarly described the need for clearer mandates and assignments for AMH [12, 13, 35–39]. The national strategy for mental health and suicide prevention suggests establishing a national coordination function for mental health and suicide prevention [12]. A national coordinator was appointed in April 2024 focused on developing and strengthening work in suicide prevention [40]. A good practice example mentioned by a respondent was the strong national governance of AMH in Norway:

*“We [in Sweden] have a structure […] that is based on a strong regionalism and a strongly developed local delegation from politics. […] Sweden can learn a lot […]. In Norway*,* you have a much stronger state governance and that means that you can decide things directly: ‘Now we do it this way’. And that possibility exists in Sweden*,* but it is less self-evident in this regional system.”* (Interview 16, male, academia).

The provision of first-line care for adolescents with mild to moderate mental health problems was one area that lacked leadership and accountability for results, according to the adolescents in the FGDs. Better coordination and quality of first-line care could help to meet the needs of adolescents and shorten queues to psychiatric care. According to the respondents, CSOs try to compensate for the lack of access to a functioning first-line care, for example, by providing telephone helplines for adolescents. Moreover, teachers, nurses, social workers and other staff at schools, primary health care centres and social services feel pressured to provide first-line care, while they may lack the knowledge, capacity, and time to do so. Good practice examples of first-line care mentioned include the *Headspace* model in Denmark, providing an adolescent-friendly ‘one-stop-shop’ service to access a range of mental health programs [41], and the 2011 health system reform in Finland, enforcing the accessibility of care within three weeks with a focus on adolescents’ needs [42].

The consultation participants emphasized the need for meaningful adolescent engagement, which was confirmed by the document review [6, 7, 43, 44]. The consultation participants argued for AMH as a human right and an issue of democracy, noting the challenge to the rights of adolescents under 18 years who are not allowed to vote and cannot fully influence decisions affecting their mental health and well-being. The participants pointed out the possibility of a whistle-blower function for adolescents, e.g., to draw decision-makers’ attention to areas that fail to meet adolescents basic AMH needs due to lack resources.

### Theme 4. weak multidisciplinary and multisector collaboration for AMH

*“It’s not like*,* for example*,* schools and social services jointly saying: ‘These are our goals and now we must work together to achieve them*,* and follow them up together.’– That’s not how people work and […] mental health suffers because it depends on several different sectors.”* (Interview 10, male, academia).

Limited multidisciplinary and multisector collaboration impede collective action on AMH, according to the respondents. The document review demonstrated that stakeholders have been calling for more multidisciplinary and multisector collaboration for many years [36, 38, 45, 46]. Silos persist within the government with unsynchronized government grants for schools, health care, and social services. Silos also exist between stakeholders with separate records, goals, plans and budgets, different legal contexts, and incentives. The silos make the system ineffective, inefficient, and difficult to navigate, especially for young people seeking care. The respondents stated that adolescents and their parents often do not know where to get help. In the absence of well-coordinated, person-centered care, families often must take coordination efforts into their own hands and try to navigate the health care system. Consequently, adolescents are sent from one provider to the next (e.g., school health care, primary care, and social services), repeatedly telling their stories but not getting the care and support that they need.

The initiatives to strengthen multidisciplinary and multisector collaboration for AMH are few and often inadequate, reported the respondents. The document review confirmed that collaborative initiatives have often been short-term and project-based, without addressing the persisting structural and organizational barriers, including the lack of time for and priority given to collaboration [35, 47, 48]. The national strategy for mental health and suicide prevention emphasizes the need for multidisciplinary and multisector collaboration for society to have a common direction in the work with mental health and suicide prevention [12]. Some CSOs collaborate with each other, while their collaboration with government agencies is limited, respondents said. The Swedish Association of Local Authorities and Regions has tried to support stronger collaboration but with limited success. Respondents suggested that joint multisector activities across administrative boundaries that include adolescents in meaningful ways, for example, by discussing real time solutions, would help strengthen collaboration and decentralize coordination. A good practice example mentioned was Västerbotten commune in Northern Sweden where psychiatric care, primary health care, schools, and social services collaborate, including through coordination of referrals [49].

The consultation participants highlighted the need for national standardized guidelines implemented across communal and regional borders, youth-centred coordination and person-centred care for adolescents. The participants also emphasized the need for structural reform and explicit funding for collaboration across sectors and disciplines, highlighting youth clinics as an example of good practice working across silos to strengthen safe places for adolescents. Running youth clinics is still voluntary for regions and communes, without and a clear mandate or mission [19], while the participants highlighted the huge potential for standardizing, expanding and strengthening youth clinics for improved AMH nationwide.

Finally, schools have a key role in multisector collaboration for AMH while their exact role is unclear, the respondents said. Various reports mention schools as one of the most important platforms to reach children and adolescents and to partner with to improve AMH outcomes nationally [6, 7, 12, 50–53]. On the one hand, the school environment was often seen as a driver of AMH challenges e.g., through performance pressure, stress, mobbing, and the choice of schools that exacerbates inequalities. On the other hand, schools were considered a platform for reaching adolescents with interventions for better health and well-being. While some schools initiate action on AMH, integrating AMH in the school curriculum and teacher training, there is a risk of overburdening schools with a responsibility without providing more dedicated resources, an adolescent explained.

*“Schools see themselves as having to deal with all societal problems - whether it’s about integration*,* solving gang violence or mental ill-health. Yes*,* schools have a lot on their plate and then you can hear a certain reluctance*,* which is more about not having time rather than not wanting to work on this [AMH].”* (Adolescent, FGD 1).

## Discussion

This study shows that the attention to AMH in Sweden has increased but not yet translated into effective financing and youth-centered collective action mainly because of (1) the limited data and evidence related to AMH; (2) the divergent definitions of and ways of framing AMH; (3) the growing but fragmented AMH stakeholder landscape; and (4) the weak multidisciplinary and multisector collaboration for AMH.

Table 2 links the four themes (main barriers) with key opportunities and possible actions, all derived from the study data, and suggests responsible stakeholders to take those actions forward. The main stakeholders include decision-makers, funders, researchers and advocates of AMH.

**Table 2:** Summary of themes, opportunities, possible actions and responsible stakeholders

Themes	Opportunities	Possible actions	Responsible stakeholders
Limited data and evidence related to AMH	Advance data and evidence on AMH, especially on what works in AMH prevention and promotion	- Coproduce data and evidence with adolescents, tracking the trends in AMH outcomes at the population level. - Evaluate the effectiveness of programs and interventions. - Improve and apply the “toolbox” for AMH data and evidence, and develop and use standardized indicators. - Hold stakeholders accountable for their obligation to report. - Use and share across sectors the available data generated from different sources, including health and social services, and schools.	Researchers
Divergent definitions of and ways of framing AMH	Deliberate and agree on a broadened definition and framing of AMH, led by youth and using a positive narrative	- Unite around a positive narrative that highlights the benefits of investing in AMH across the mental health continuum. - Use clear definitions and relevant terminology to foster a common, more nuanced language around mental health. - Continue to fight stigma directed towards adolescents with mental health challenges, especially related to suicidal ideation and suicide which remain highly stigmatized.	All stakeholders, especially advocates of AMH
Growing but fragmented AMH stakeholder landscape	Strengthen leadership, accountability and adolescent engagement for AMH	- Develop a national plan specifically focused on AMH, with AMH reflected in budget allocations across sectors and administrative levels, and clarity on roles, mandates and lines of accountability. - Improve leadership for the coordination and quality of first-line care for adolescents with mild to moderate mental health problems, and investment in youth-centered services at primary care level. - Foster meaningful adolescent engagement and leadership in the growing AMH stakeholder landscape, especially among national level stakeholders.	Decision-makers, funders, and researchers
Weak multidisciplinary and multisector collaboration for AMH	Break the silos and enhance multidisciplinary and multisector collaboration for AMH	- Develop standardized guidelines across communal and regional borders, and implement person-centred care. - Implement structural reform to enable stronger coordination, especially at the level of service interaction with youth. - Incentivize collaboration across sectors and disciplines, including through explicit funding. - Implement joint multisector activities across administrative boundaries that include adolescents in meaningful ways, e.g., by discussing real time solutions. - Engage school as one of the most important platforms to reach children and adolescents and to partner with to improve AMH outcomes nationally.	Decision-makers, funders and researchers

Implementation of the suggested actions by responsible stakeholders, including youth, would enable the translation of national policies and strategies into more effective financing and youth-centered collective action.

### Strengths and limitations

Strengths: Triangulation of data from various sources; engagement of adolescents and young adults throughout the research process; validation of findings through consultations; and, adaptation of a political science framework that draws on social science theory pertaining to collective action, providing a useful analytical tool to explore the barriers for AMH financing and action.

Limitations: Conducting the interviews and FGDs via Zoom instead of face-to-face; getting the perspectives from only a few adolescents with lived experience of mental ill-health and none between 10 and 14 years of age; and, engaging only stakeholders based in Stockholm in the consultation.

## Conclusions

Transformational change is needed to improve AMH outcomes in Sweden through effective financing across sectors in support of youth-centred collective action. Identified barriers may be overcome by: researchers focusing on advancing data and evidence, especially on what works in AMH prevention and promotion; stakeholders, especially advocates of AMH, deliberating and agreeing on a broadened definition and framing of AMH, led by youth and using a positive narrative that highlights the benefits of investing in AMH for individuals and society at large; decision-makers, funders and researchers strengthening leadership, accountability and adolescent engagement for AMH, and enhancing multidisciplinary and multisector collaboration for AMH, backed up by well-coordinated, youth-centered health and social services, learning from good practices within and outside of Sweden.

## Data Availability

This study is based on a sample of key-informants and focus group participants who are well-known in the field of AMH; making the full data set publicly available would breach their privacy. The informed consent that all respondents signed promised full anonymity. Following data requests, interview and focus group transcripts will be reviewed for any potential identifying information and will only be made available to researchers who sign a data sharing agreement.

## Abbreviations

AMH: Adolescent mental health and well–being
COVID-19: Coronavirus disease 2019
CSO: Civil society organization
FGD: Focus group discussion
LGBTQIA+: Lesbian, gay, bisexual, transgender, queer, intersex, and asexual
MMAPP: Measuring Mental Health Among Adolescents and Young People at the Population Level
UNICEF: United Nations Children’s Fund
